# Plasmapheresis for the treatment of thyroid storm

**DOI:** 10.1530/EDM-25-0010

**Published:** 2025-10-13

**Authors:** Luis Miguel Osorio-Toro, Yessica Alejandra Ordoñez-Guzman, Jhon Herney Quintana-Ospina, Mónica Lucía Vergara-Portocarrero, Edwin Alexander Lizarazo-Herrera, Jorge Enrique Daza-Arana, María Angelica Rodríguez-Scarpetta, Katherine Restrepo-Erazo, Andrés Felipe García Ramos

**Affiliations:** ^1^ Specialization in Internal Medicine, Department of Health, Universidad Santiago de Cali, Cali, Colombia; ^2^ Department of Research and Education, Clínica de Occidente S.A., Cali, Colombia; ^3^ Genetics, Physiology, and Metabolism Research Group (GEFIME), Universidad Santiago de Cali, Cali, Colombia; ^4^ Health and Movement Research Group, Universidad Santiago de Cali, Santiago de Cali, Colombia

**Keywords:** thyroid crisis, hyperthyroidism, thyroid gland, plasmapheresis, therapeutics

## Abstract

**Summary:**

Thyroid storm, also known as thyroid crisis, is a serious medical condition that occurs when there is an extreme overproduction of thyroid hormones. It usually develops in individuals with uncontrolled hyperthyroidism, often due to diseases such as Graves' disease or thyroid adenomas. We herein report a case of a female patient with Graves' disease who presented with thyroid storm and did not respond to conventional treatment, requiring intensive care unit management and mechanical ventilation support. In addition, she was managed with plasma exchange (plasmapheresis), which stabilized her clinical and biochemical parameters. In conclusion, thyroid storm is a critical condition with multiple clinical implications that should be managed using a multidisciplinary approach; moreover, early identification and adequate treatment are essential to reduce its associated morbidity and mortality. Our case indicated that plasmapheresis should be considered for patients refractory to conventional treatment. Once the critical stage of the disease concludes, definitive treatment with total thyroidectomy should be planned.

**Learning points:**

- Early recognition and prompt management of thyroid storm can significantly improve patient outcomes.
- Multidisciplinary care is essential for addressing the systemic effects of thyroid storm.
- Tailored rehabilitation programs may enhance recovery from associated complications, such as paralysis.

## Background

Thyroid storm is a rare endocrine emergency condition and is associated with a high mortality rate. The incidence of thyroid storm has been reported to be 0.20–0.76 per 100,000 individuals per year, with an incidence of 4.8–5.6 per 100,000 hospitalized patients (1), with few reports from Colombia (2, 3). Thyroid storm was first defined in 1926 as an exophthalmic goiter crisis and was considered an exacerbation of Graves' disease with excessive hyperthyroidism. Although thyroid storm is most common in patients with untreated or uncontrolled Graves' disease, it can also occur in solitary toxic adenomas or toxic multinodular goiter. It can also be precipitated by trauma, infection, amiodarone use, parturition, acute iodine load, thyroid or non-thyroid surgery, and failure to take antithyroid medication (4, 13).

In this context, it is important to differentiate between hyperthyroidism and thyrotoxicosis, which are often mistakenly confused. Hyperthyroidism is the result of excessive synthesis and secretion of thyroid hormone from the thyroid gland, whereas thyrotoxicosis is characterized by high levels of T3 and T4 in peripheral tissues regardless of the source and can range in severity from subclinical thyrotoxicosis to overt thyrotoxicosis. Its most severe presentation implies severe end-organ dysfunction and is known as thyroid storm, as presented in our case (5).

In thyroid storm, exaggerated hyperthyroidism symptoms such as tachycardia, dyspnea, diarrhea, fever, and even death may occur. Standard treatment is based on comprehensive management in the intensive care unit (ICU) with beta-blockers, thionamides, corticosteroids, Lugol, and cholestyramine, which aid in achieving disease control in most patients (6, 7). In cases where thyrotoxicosis is refractory to conventional therapy or conventional therapy is not tolerated, plasmapheresis should be considered as an alternative treatment strategy (8, 9, 10).

Here, we report the case of a patient with Graves' disease who developed thyroid storm. As she was refractory to conventional treatment, therapeutic plasma exchange (also known as plasmapheresis) was used, which helped in achieving both clinical and biochemical stabilization.

## Case presentation

Our patient was a 27-year-old woman with a history of discoid lupus erythematosus and Sjogren’s disease who was undergoing outpatient follow-up by the endocrinology unit for an 8-month history of Graves' disease. She was being treated with methimazole (40 mg daily) and propranolol (40 mg every 12 h) and was scheduled for outpatient radioiodine therapy, but in the last 7 days the patient abandoned treatment without just cause. She presented to the emergency department with a 3-day history of asthenia, adynamia, diarrhea, unquantified fever spikes, agitation, palpitations, and dyspnea on mild exertion.

## Investigation

Upon admission to the emergency room, she presented a Burch and Wartofsky scale score of 60 points, given temperature 38.5°C, tachycardia 125 beats per minute, diarrhea, agitation, and a precipitating event due to discontinuation of medication, associated with tests (Table 1) consistent with thyroid storm due to Graves' disease. As a result, ICU management was indicated with methimazole (80 mg daily), propranolol (80 mg every 4 h), Lugol (five drops every 6 h), hydrocortisone (300 mg bolus and 100 mg every 8 h), and cholestyramine (4 g every 6 h).

**Table 1:** Laboratory studies on admission.

Parameters	Result	Reference range
Creatinine, mg/dL	0.61	0.5–1.1
Leukocytes, ×10^9^/L	5,730	4.0–11.8
Hemoglobin, g/dL	11.2	12.3–15.3
Platelets, cells/mm^3^	265,000	203,000–445,000
AST, U/L	30	8–33
ALT, U/L	25	4–36
Thyrotropin, µIU/mL	0.002	0.35–5.50
Free T4, ng/dL	6.31	0.8–1.7
Free T3, pg/mL	>20	1.8–4.6
Anti-TSH receptor antibodies, IU/L	37.2	0–1.75

TSH, thyroid-stimulating hormone; AST, aspartate aminotransferase; ALT, alanine aminotransferase.

## Treatment

**Figure 1:**
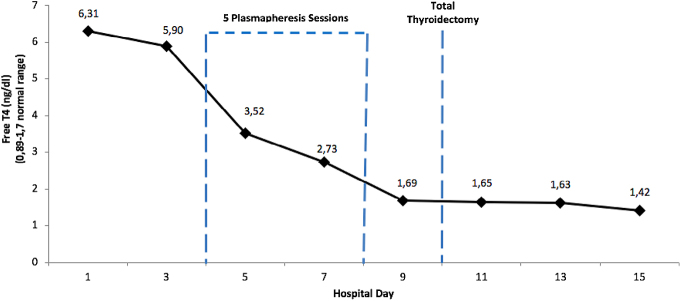
Evolution of free T4 by days of hospital stay.

The patient had a slow and complicated course, requiring mechanical ventilation support on day 2 due to ventilatory failure. By day 3 of comprehensive ICU management, control of the disease was not achieved, and the patient still had high free T4 levels, unstable clinical parameters, and a Burch and Wartofsky score within the thyroid storm range. As a result, the endocrinology and nephrology teams decided to start plasmapheresis, administered daily with 4% albumin as plasma replacement until systemic symptoms were controlled, which was achieved after five sessions. Following this therapy, hemodynamic control was achieved, mechanical ventilation was discontinued, and free T4 levels, measured every 48 h, decreased to normal values. Consequently, definitive treatment with total thyroidectomy was performed (Fig. 1). The patient evolved steadily, requiring management of hypothyroidism with 200 μg of levothyroxine and temporary calcium and calcitriol supplementation to manage postsurgical hypoparathyroidism, which was subsequently resolved. The patient was eventually discharged, and during outpatient follow-up, she remained clinically stable with an acceptable quality of life.

## Outcome and follow-up

The patient was discharged following stabilization of thyroid hormone levels and comprehensive multidisciplinary care. A tailored rehabilitation program addressed her paralysis, leading to a gradual recovery of motor function. During outpatient follow-up, she remained clinically stable, with thyroid function tests within normal limits and no recurrence of symptoms. Regular endocrinological assessments and physical therapy have maintained her acceptable quality of life.

## Discussion

Thyroid storm is an endocrine emergency condition characterized by severe expression and exacerbation of the symptoms of thyrotoxicosis. A patient with thyroid storm has up to 12 times higher mortality than patients with nonaggravated hyperthyroidism (11). The diagnosis of thyroid storm is clinical, based on the presence of severe, life-threatening symptoms such as cardiovascular dysfunction, altered mental status, and hyperpyrexia in patients with biochemical evidence of hyperthyroidism with undetectable or low thyrotropin values associated with high free T4 and/or free T3 levels (12), as in our case. Thyroid storm can occur in the context of toxic multinodular goiter and solitary toxic adenomas; although the most frequent condition in which it occurs is Graves' disease and it can be precipitated by trauma, infection, amiodarone use, parturition, acute iodine load, thyroid or non-thyroid surgery, and failure to take antithyroid medication (13). Our patient presented with Graves' disease, which was confirmed by the presence of positive anti-TSH receptor antibodies.

The treatment of thyroid storm aims at controlling thyrotoxicosis and managing the triggering factor by blocking thyroid hormone synthesis, its release from the gland, peripheral conversion of T4 to T3, peripheral circulation of thyroid hormone, and beta-adrenergic activity. Antithyroid drugs, glucocorticoids, β-adrenergic blockers, inorganic iodide, and cholestyramine are generally used for the treatment of thyroid storm (14). Regarding the preferred antithyroid drug, US guidelines favor the use of propylthiouracil since this drug, unlike methimazole, blocks the conversion of T4 to T3 (14). However, a recent publication found no difference in hospital mortality between those treated with propylthiouracil and those treated with methimazole (15). In nonresponders, plasmapheresis may be an effective and safe option (3).

Therapeutic plasmapheresis as a therapy for thyroid storm was first described in 1970 (16). Plasmapheresis removes cytokines, antibodies, and plasma thyroid hormones, stabilizing the clinical condition of a patient. The American Society of Apheresis currently recommends plasmapheresis every 1 to 3 days in patients with thyroid storm until clinical stabilization is achieved. In severe cases where the patient does not improve within 48 h after first-line treatment, or if there is a contraindication, a 2C grade of plasmapheresis is recommended (17). Serious adverse effects of plasmapheresis include anaphylaxis, coagulopathy, vascular injury, and seizures, but they are generally well tolerated, as in our case (12, 18). The effect of plasmapheresis is transient and helps prepare the patient for definitive therapy, such as surgical resection. Some reports indicate a 21% reduction in thyroid hormone levels after each treatment, reaching up to 55% after four sessions (19). Another retrospective study involving 46 patients with thyroid storm who required plasmapheresis found a reduction of up to 45% in free T4 levels after four sessions in both patients with Graves' disease and those with other thyrotoxicosis as the underlying cause (20). In our case, plasmapheresis was indicated 72 h after the beginning of conventional therapy and required five sessions to achieve clinical and biochemical stabilization. Following this, definitive treatment with total thyroidectomy was performed.

In conclusion, thyroid storm is a life-threatening endocrine emergency condition with a significant mortality rate that requires high clinical suspicion and immediate treatment to achieve clinical stabilization. Plasmapheresis should be considered for patients refractory to conventional treatment. Once the critical condition subsides, definitive treatment with total thyroidectomy should be planned.

## Declaration of interest

The authors declare that there is no conflict of interest that could be perceived as prejudicing the impartiality of the work reported.

## Funding

This investigation was funded by the Dirección General de Investigaciones de la Universidad Santiago de Cali under call No. DGI-01-2025 and project DGI- 442-621123-232.

## Patient consent

The patient signed a consent form for the publication of this case report.

## Author contributions

LMOT, YAOG, JHQO, MARS, and MLVP were responsible for conceptualization. LMOT, EALH, and JEDA handled writing. LMOT, KRE, AFGR, and YAOG conducted the investigation. EALH and JEDA managed visualization. KRE and AFGR performed validation. LMOT, JEDA, and MARS were responsible for review and editing. JHQO and LMOT provided supervision.
